# Genome-wide detection of copy number variations in polled yak using the Illumina BovineHD BeadChip

**DOI:** 10.1186/s12864-019-5759-1

**Published:** 2019-05-14

**Authors:** Congjun Jia, Hongbo Wang, Chen Li, Xiaoyun Wu, Linsen Zan, Xuezhi Ding, Xian Guo, Pengjia Bao, Jie Pei, Min Chu, Chunnian Liang, Ping Yan

**Affiliations:** 1 Key Laboratory of Yak Breeding Engineering Gansu Province, Lanzhou Institute of Husbandry and Pharmaceutical Sciences, Chinese Academy of Agricultural Sciences, Lanzhou, 730050 China; 2 College of Animal Science and Technology, Northwest A&F University, Yangling, 712100 China

**Keywords:** Polled yak, Copy number variation, Illunima BovineHD BeadChip, High altitude adaptation, Economic traits

## Abstract

**Background:**

Copy number variations (CNVs), which are genetic variations present throughout mammalian genomes, are a vital source of phenotypic variation that can lead to the development of unique traits. In this study we used the Illunima BovineHD BeadChip to conduct genome-wide detection of CNVs in 215 polled yaks.

**Results:**

A total of 1066 CNV regions (CNVRs) were detected with a total length of 181.6 Mb, comprising ~ 7.2% of the bovine autosomal genome. The size of these CNVRs ranged from 5.53 kb to 1148.45 kb, with an average size of 170.31 kb. Eight out of nine randomly chosen CNVRs were successfully validated by qPCR. A functional enrichment analysis of the CNVR-associated genes indicated their relationship to a number of molecular adaptations that enable yaks to thrive at high altitudes. One third of the detected CNVRs were mapped to QTLs associated with six classes of economically important traits, indicating that these CNVRs may play an important role in variations of these traits.

**Conclusions:**

Our genome-wide yak CNV map may thus provide valuable insights into both the molecular mechanisms of high altitude adaptation and the potential genomic basis of economically important traits in yak.

**Electronic supplementary material:**

The online version of this article (10.1186/s12864-019-5759-1) contains supplementary material, which is available to authorized users.

## Background

Genomic variations in terms of both sequence, as in the case of single nucleotide polymorphism base substitutions, and structure, as in instances of insertions, deletions, inversions, and copy number variations, can lead to the development of a diverse range of phenotypes that can be of economic importance when present in livestock. Copy number variations (CNVs) are a type of genome structure variation in which genomic segments ranging from 50 bp to several Mb are affected by large-scale insertions, deletions, duplications, inversions, and translocations relative to the reference genome [1]. There are four primary mechanisms through which CNVs form: non-allelic homologous recombination (NAHR), non-homologous end-joining repair of double-stranded breaks (NHEJ), fork stalling and template switching (FoSTeS) and retro-transposition [2, 3]. These CNVs are a major source of genetic variation [2, 4], and can cause substantial phenotypic variations through a variety of mechanisms including a gene dosage effect, changes in gene expression level, a gene blocking effect, gene fusion, a positional effect, expression of previously silent alleles, functional polymorphisms, and potential condensed effects [5]. As a common form of structural variation in vertebrate genomes, functional studies of CNVs have been conducted in many organisms. In human, CNVs are associated with many common diseases [6–8], including cancer [9] and autoimmunity [10]. In cattle, CNVs are correlated with parasite resistance [11], growth traits [5, 12], reproduction [13, 14], milk production and composition [15, 16], milk somatic cell scores [17], meat quality [18, 19], and feed conversion ratios [20]. In goats, CNVs in Agouti Signaling Protein (*ASIP*) gene have identified it as a genetic factor which is associated with coat colors in different breeds [21]. In pigs, CNV regions harboring candidate genes responsible for many complex traits related to meat production and meat quality have been identified [22]. Sex-determining region Y-box 5 gene (*SOX5*) and *SOX6* CNVs are also known to be relevant to Pea-comb and muscle development in chickens, respectively [23, 24]. CNVs in *SOX6* promote the proliferation and differentiation of skeletal muscle cells as elevated *SOX6* expression drives the up-regulated expression of muscle-growth-related genes in chickens [24].

The yak (*Bos grunniens*) lives on the Qinghai-Tibetan Plateau (QTP) – an extremely cold, harsh, and oxygen-poor ecosystem – where it serves as an essential resource for Tibetans and other nomadic peoples in this region [25]. The yak is the primary form of livestock for the inhabitants of QTP, making its economic importance clear. Despite this importance, efforts to improve the genetics of the domestic yak lag far behind those in beef cattle, dairy cattle, and other livestock. While specific genetic-based efforts made in this context are limited, as a mammal native to this highland region the yak has developed comprehensive adaptations that equip it to survive in this harsh environment [26, 27]. As such, it is an ideal model organism to study in order to explore the formation and mechanisms of high altitude adaptation. From an evolutionary and domestication perspective, the yak also represents a unique genetic resource as it is believed to be the only large animal coexisting with its wild ancestors in a similar environment [28]. A comprehensive understanding of yak genetics thus offers an opportunity to not only accelerate genetics-based yak breeding efforts, but also to understand the molecular basis of high altitude adaptations and other unique traits in these animals.

To date only one yak CNV map is available [27], which is far less than is available for other species of livestock. Since the first three cattle CNV maps were published in 2010 [29–31], numerous additional genome-wide CNV maps have been constructed either using SNP arrays [32–34] or next-generation sequencing [16, 35, 36]. Similarly, in sheep there are now several genome-wide CNV maps available [37, 38] since the first CNV map publication in 2011 [39]. Given the dearth of yak CNV maps, and the fact that a significant proportion of phenotypic variation is believed to be accounted for by CNVs throughout mammalian genomes, there is thus a clear and urgent need to establish a comprehensive atlas of CNVs in the yak genome.

Currently, three major methods, namely array comparative genomic hybridization (CGH), SNP arrays, and next-generation sequencing (NGS), have been used to detect genome-wide CNVs in livestock, as in cattle. SNP arrays were proposed to be advantageous relative to CGH and NGS due to their lower prices, lower signal-to-noise ratios, and the use of the parameter B-allele frequency which facilitates result interpretation [40]. In addition, SNP arrays can also provide a relatively high resolution of CNVs, although not at a level comparable to that provided by NGS, and these arrays are more convenient for high-throughput analyses and genome-wide association studies due to their quantification of allele-specific copy numbers [41]. The aforementioned yak CNV map was conducted by using an NGS-based method, and it provided valuable data for understanding yak domestication and adaptations to living in a high-altitude environment [27]. The SNP array-based method, however, has not been reported for yak genome CNV detection yet. Thus, considering all the advantages of these SNP arrays and the follow-up large-scale population studies including CNV-based genome-wide association studies, it is of value to evaluate CNVs throughout the yak genome using SNP arrays.

Polled yak is a new easy-to-manage breed of yak bred by scientists from the Lanzhou Institute of Husbandry and Pharmaceutical Sciences of Chinese Academy of Agricultural Sciences (CAAS) in China. In the present study, we conducted a genome-wide CNV mapping effort in yaks using SNP data from 215 polled yaks. To the best of our knowledge, this is the first yak CNVR map constructed based on Illumina BovineHD BeadChip genotype data. The objective of this study was to construct a genome-wide CNVs map of polled yaks using a SNP genotyping method in order to facilitate genetic improvements in yaks and to explore the genetic basis of high-elevation adaptation.

## Results

### Genome-wide detection of CNVs and CNVRs

We identified 39,388 autosomal CNVs in these 215 polled yaks, among which 37,009 CNVs were losses (deletions) and 2379 CNVs were gains (duplications) (Additional file 1: Table S1). The ratio of loss events to gain events was 15.56:1. On average, each yak had 183.2 CNVs across its genome.

**Fig. 1:**
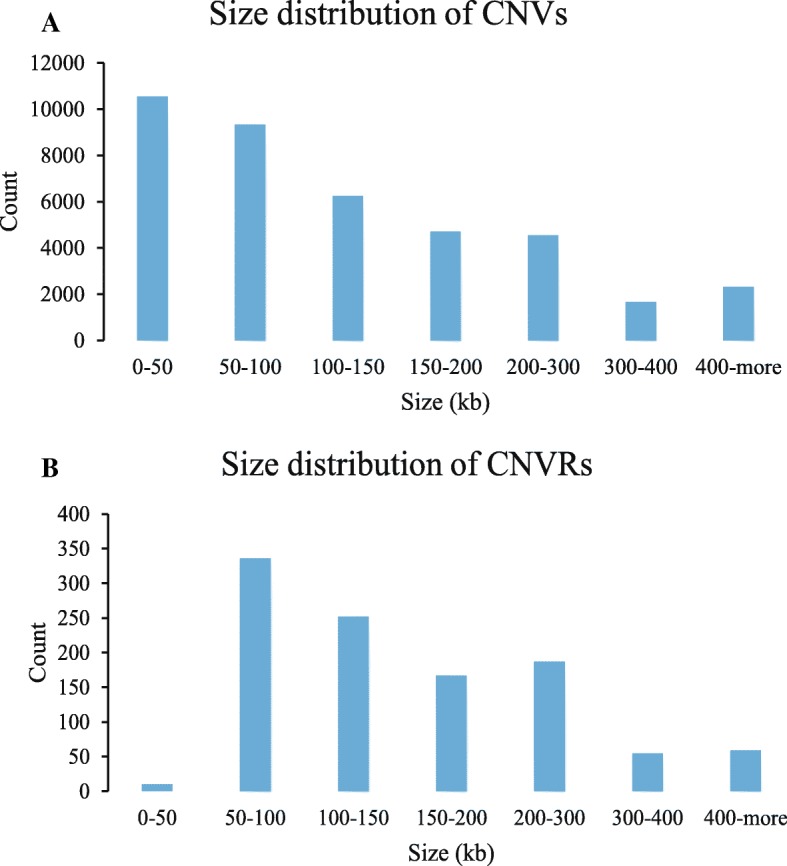
Size distribution of CNVs (**a**) and CNVRs (**b**) in polled yaks

The size of the identified CNVs ranged from 0.697 kb to 4906.179 kb, comprising 3 to 1204 SNP markers. The mean size of these CNVs was 140 kb and the median length was 98.745 kb. The distribution of CNV sizes is presented in Table 1 and Fig. 1a. Almost half of the CNVs ranged from 0 to 100 kb in size, with 26.78% being 0–50 kb and 23.71% being 50–100 kb in length, respectively. CNVs with sizes 300–400 kb or larger than 400 kb were relatively rare.

**Table 1:** CNV and CNVR size distributions

Size (kb)	Number of CNVs	Percentage %	Number of CNVRs	Percentage%
0–50	10,547	26.78	10	0.94
50–100	9342	23.72	336	31.52
100–150	6258	15.89	252	23.64
150–200	4707	11.59	167	15.67
200–300	4552	11.56	187	17.54
300–400	1670	4.24	55	5.16
> 400	2312	5.87	59	5.53

Before merging the overlapping CNVs across all individuals to identify CNVRs, we filtered raw CNV calling results to retain only those containing > 10 SNPs and with a size > 50 kb. After filtering, 27,200 CNVs remained for further analysis (Additional file 2: Table S2). After merging the overlapping CNVs using the *CNVRuler* [42] software, a total of 1066 CNVRs were obtained (Additional file 3: Table S3).

Table 1 and Fig. 1b summarize the size distribution of these CNVRs. The majority of these CNVRs (31.52%) ranged from 50 to 100 kb in size, while 23.64% were between 100 and 150 kb. Overall, these CNVRs ranged from 5.53 kb to 1148.45 kb, with an average size of 170.31 kb.

**Fig. 2:**
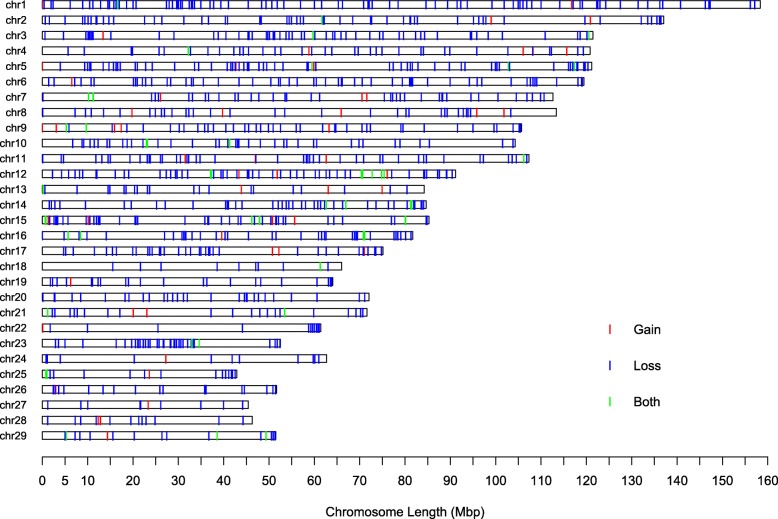
Genome-wide CNVR map in polled yak. Red, blue and green represent gain events, loss events, and both gain and loss events, respectively

Among the 1066 identified CNVRs, 55 were gain events, 959 were loss events, and 52 CNVRs were composed of both gain and loss events. Distributions of autosomal CNVRs are presented in Table 2 and Fig. 2. The total length of the identified CNVRs was 181.55 Mb, occupying 7.23% of the bovine genome (UMD_3.1). The number of CNVRs per chromosome varied from 10 on chr 18 and chr 27 to 87 on chr 1. The percentage of CNVR as a fraction of overall chromosome length ranged from 2.56% on chr 22 to 11.91% on chr 12.

**Table 2:** The distribution of CNVRs in the yak genome (based on UMD_3.1)

Chr	No. of CNVRs	Length of CNVRs (bp)	Length of chr (bp)	Percentage (%)
1	87	14,733,639	158,337,067	9.31
2	57	11,349,756	137,060,424	8.28
3	57	9,733,648	121,430,405	8.02
4	42	7,735,084	120,829,699	6.4
5	69	13,030,532	121,191,424	10.75
6	57	9,933,389	119,458,736	8.32
7	44	6,224,814	112,638,659	5.53
8	37	5,417,670	113,384,836	4.78
9	46	8,805,118	105,708,250	8.33
10	41	7,414,048	104,305,016	7.11
11	49	7,910,231	107,310,763	7.37
12	60	10,854,724	91,163,125	11.91
13	25	3,352,203	84,240,350	3.98
14	43	8,562,586	84,648,390	10.12
15	51	6,923,797	85,296,676	8.12
16	40	6,421,950	81,724,687	7.86
17	38	6,835,771	75,158,596	9.1
18	10	2,224,179	66,004,023	3.37
19	23	3,684,893	64,057,457	5.75
20	29	6,019,214	72,042,655	8.36
21	24	3,365,086	71,599,096	4.7
22	12	1,572,517	61,435,874	2.56
23	38	5,611,441	52,530,062	10.68
24	12	1,668,878	62,714,930	2.66
25	16	2,529,243	42,904,170	5.9
26	18	3,193,189	51,681,464	6.18
27	10	1,715,702	45,407,902	3.78
28	14	2,331,191	46,312,546	5.03
29	17	2,396,170	51,505,224	4.65
Total	1066	181,550,663	2,512,082,506	7.23

### Validation of CNVRs by qPCR

**Fig. 3:**
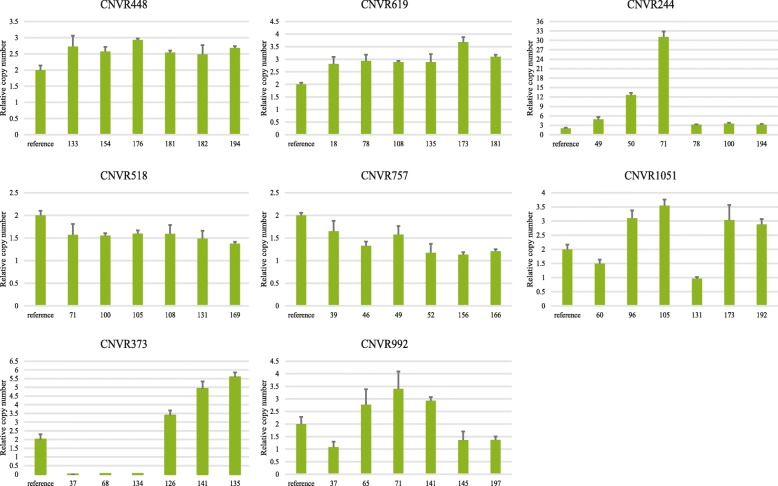
qPCR results for 8 validated CNVRs. The y-axis shows the normalized ratios and x-axis shows the references and samples. Samples with normalized ratios of appropriately 0 or 1 represent individuals with instances of copy number loss, while those with values of appropriately 3 or more represent individuals with copy number gains (*P* < 0.05). Values of 2 represent a normal copy number

Nine CNVRs were randomly chosen to confirm the reliability of the identified CNVRs. The chosen CNVRs represented three types of CNVs with frequencies ranging from low to high. The results of a validation qPCR confirmed that eight of these CNVRs (88.9%) were representative of true genomic variations (Fig. 3). The ninth was CNVR339, which was a loss-type CNV. The qPCR result for this region showed that there was no copy number loss in the samples tested, and all samples showed a copy number of two in this region.

### CNVRs annotation and enrichment analysis

**Fig. 4:**
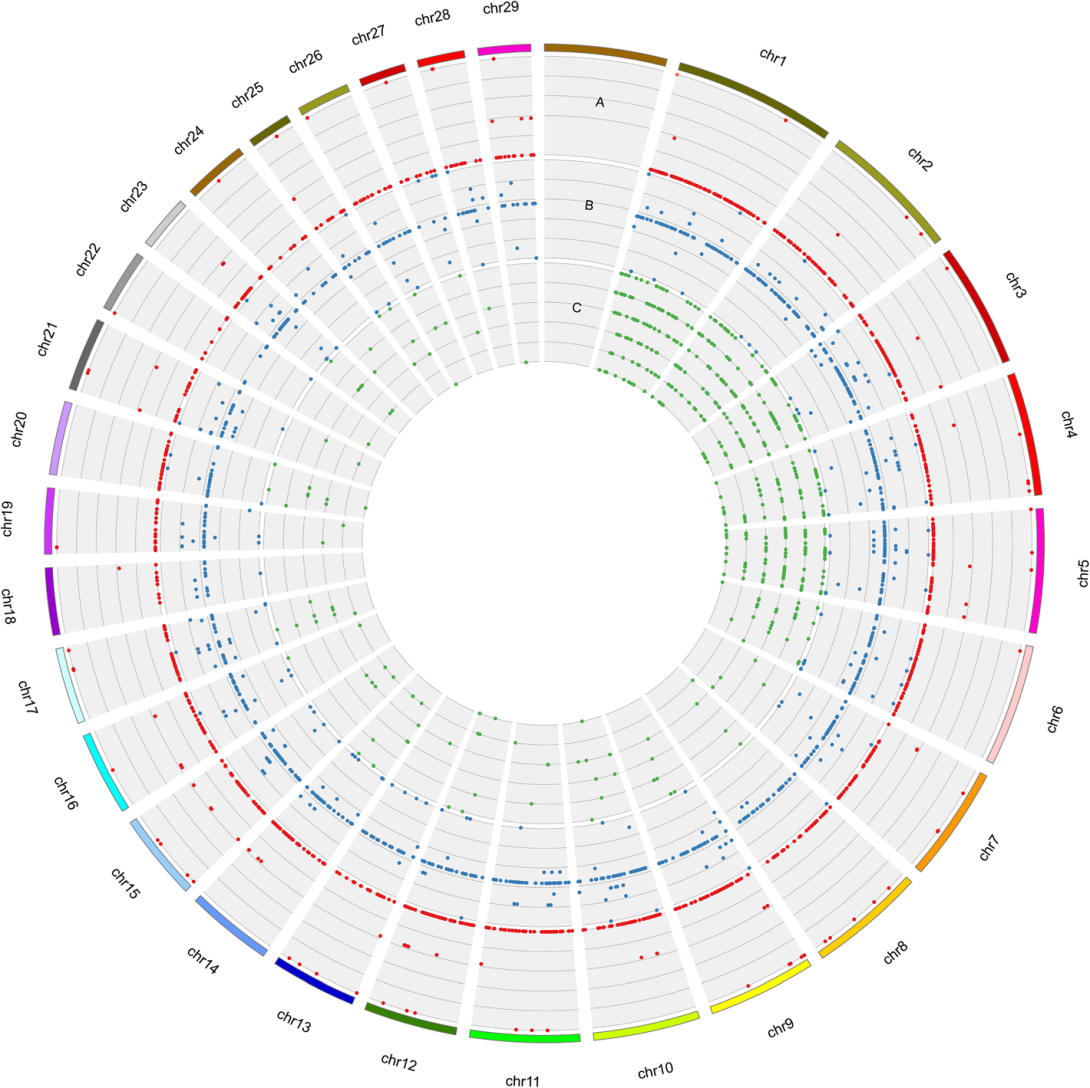
Circos plot of CNVR distributions (**a**), harbored genes (**b**), and QTLs (**c**). Red plots on different tracks, from outside to inside, in circle A represent gain, both gain and loss, and loss events, respectively. Blue plots, from outside to inside in circle B, represent rRNA, snoRNA, snRNA, protein coding genes, pseudogenes, processed pseudogenes, miscRNA, and miRNA, respectively. Green plots in circle C represent reproduction QTLs, production QTLs, milk QTLs, meat and carcass QTLs, health QTLs, and exterior QTLs, respectively

The BioMart tool was employed to map genes to the 1066 identified CNVRs. In total, 2111 genes overlapping with 768 CNVRs (72.05% of the total CNVRs) were identified. These 2111 genes were classified into eight gene types, including 1746 protein coding genes, 54 pseudogenes, 6 processed pseudogenes, 84 miRNA genes, 13 miscRNA genes, 33 rRNA genes, 84 snoRNA genes, and 91 snRNA genes (Additional file 4: Table S4, Fig. 4b). The number of genes overlapping with gain, loss and combined gain/loss type CNVRs were 86, 1854, and 171, respectively. Since the yak population used here was comprised of 215 polled yaks, we checked the genes harbored on chr 1. Interestingly, we found that three previously reported candidate genes for polled phenotype in yak [43] were harbored in CNVR3.

To further investigate the functions of genes harbored in these different types of CNVRs, an enrichment analysis of protein coding genes was performed independently on the three gene sets associated with gain, loss and both CNVRs (Additional file 5: Table S5). Both gene ontology annotation and Reactome pathway assessments were implemented for each enrichment analysis.

Genes overlapping with the CNV gain regions were significantly enriched in GO terms associated with lipid transport, sensory perception of chemical stimulus, sensory perception of smell, and cellular processes. A Reactome pathway analysis indicated that these genes were similarly enriched for olfactory signaling pathways, mitochondrial fatty acid beta-oxidation, metabolism of lipids, synthesis of phosphatidylcholine (PC), digestion and absorption, and FMO oxidizes nucleophiles. For gene sets composed of genes present in CNV loss regions, the GO enrichment analysis showed that these genes were primarily linked to the biological processes of nuclear transport, mitosis, and protein localization. Genes identified in the CNVRs containing both gain and loss events were linked to sensory perception of smell, fatty acid metabolism, JAK-STAT signaling, regulation of biological processes, G-protein coupled receptor signaling, and responses to stimulus. A Reactome pathway analysis of these same genes highlighted an enrichment for genes associated with the olfactory signaling pathway, the prolactin receptor signaling pathway, the growth hormone receptor signaling pathway, and the transport of fatty acids.

To estimate the potential effects of CNVRs involved in hypoxic response, the genes harbored in the identified CNVRs were queried in the hypoxia-related genes database, HypoxiaDB [44]. One hundred and sixty seven genes (11.6% of identified named protein-coding genes) were found in the HypoxiaDB Database gene list of genes which alter their expression under hypoxic conditions (Additional file 6: Table S6). Among these, 57 genes were up-regulated under hypoxic stress, and 84 were down-regulated. For 12 of our identified genes, shifts in expression were variable depending on the degree of hypoxia, while information of the expression of the remaining 14 genes was not available.

### QTLs overlapping with identified CNVRs

Three hundred and ninety six CNVRs (37.2%) were identified to overlap with 1736 QTLs involved in six trait classes (Additional file 7: Table S7, Fig. 4c), including reproduction QTLs (31.3%), production QTLs (13.8%), meat and carcass QTLs (8.5%), milk QTLs (32.8%), exterior QTLs (7.1%), and health QTLs (6.5%). The number of gain, loss, and both gain/loss regions overlapping with these QTLs were 14, 368, and 14, respectively. The number of traits involved in each QTL category were 33, 29, 35, 59, 26, and 15 for QTLs associated with reproduction, production, meat and carcass, milk, exterior, and health, respectively. For production QTLs, the majority of loci were associated with body weight across different life stages. For health-associated QTLs, the majority were associated with bovine tuberculosis susceptibility. QTLs pertaining to meat and carcass were mainly associated with lean meat yield, shear force, and longissimus muscle area.

### Comparison with previous studies of bovine CNVRs

The results of a comparison of our results with 31 previously reported bovine CNVR studies are summarized in Table 3. These previous studies reported total numbers of CNVRs ranging from 19 to 4562, with lengths ranging from 4.4 Mb (0.18% of the bovine genome) to 1078.5 Mb (42.9%). The number of CNVRs overlapping with our results ranged from 3 to 736. The percentage of the overlapping CNVRs per study ranged from 8.0 to 48.1%.

**Table 3:** Comparison of our study with 31 previous bovine CNV studies conducted using various platforms

Study	Platform	Breed	Sample	CNVR count	CNVR length(percentage)	Overlapping CNVR count with present study	Overlapping percentage
Liu et al., 2010 [31]	CGH	17	90	200	36.2 Mb (1.4%)	41	20.5%
Fadista et al., 2010 [30]*	CGH	4	20	224	9.4 Mb (0.37%)	18	8.0%
Kijas et al., 2011 [45]*	CGH	3	9	19	4.4 Mb (0.18%)	3	15.8%
Zhang et al., 2014 [46]*	CGH	14	27	339	32.8 Mb (1.3%)	46	13.6%
Zhang et al., 2015a [47]*	CGH	12	24	275	21.7 Mb (0.86%)	36	13.1%
Bickhart et al., 2016 [48]	CGH	8	75	1853	87.5 Mb (3.1%)	240	13.0%
Bae et al., 2010 [29]*	50 K	1	265	359	62.7 Mb (2.5%)	66	18.4%
Hou et al., 2011 [32]	50 K	21	521	682	139.8 Mb (5.5%)	209	30.6%
Hou et al., 2012b [11]*	50 K	1	472	811	130.0 Mb (5.2%)	185	22.8%
Jiang et al., 2012 [49]*	50 K	1	2047	96	23.9 Mb (0.95%)	37	38.5%
Wang et al., 2015 [50]	50 K	1	492	334	51.3 Mb (2.0%)	80	24.0%
Gurgul et al., 2015 [51]	50 K	2	1160	106	176.6 Mb (7.0%)	51	48.1%
Hou et al., 2012a [33]	BovineHD	27	674	3438	146.9 Mb (5.8%)	542	15.8%
Jiang et al., 2013 [41]	BovineHD	1	96	367	42.7 Mb (1.6%)	94	25.6%
Wu et al., 2015 [52]	BovineHD	1	792	263	35.5 Mb (1.4%)	53	20.2%
Zhang et al., 2015b [53]	BovineHD	1	6	365	13.1 Mb (0.52%)	65	17.8%
da Silva et al., 2016a [15]	BovineHD	1	1509	4097	1078.5 Mb (42.9%)	736	18.0%
da Silva et al., 2016b [18]	BovineHD	1	723	2649	170.6 Mb (6.8%)	546	20.6%
Sasaki et al., 2016 [54]	BovineHD	1	791	861	43.7 Mb (1.7%)	189	22.0%
Xu et al., 2016 [55]	BovineHD	8	300	263	15.6 Mb (0.62%)	40	15.2%
Zhou et al., 2016a [56]	BovineHD	1	2230	231	70.4 Mb (2.8%)	57	24.7%
Zhou et al., 2016b [57]	BovineHD	1	1682	4562	186 Mb (7.5%)	629	13.8%
Prinsen et al., 2016 [58]	BovineHD	1	1410	563	57.6 Mb (2.3%)	112	19.9%
Upadhyay et al., 2017 [34]	BovineHD	38	149	923	61.1 Mb (2.4%)	224	24.3%
Yang et al., 2017 [59]	BovineHD	8	167	3356	148.0 Mb (5.8%)	574	17.1%
Karimi et al., 2018 [60]	BovineHD	1	90	221	36.4 Mb (1.4%)	59	26.7%
Stothard et al., 2011 [35]*	Resequencing	2	2	693	3.8 Mb (0.15%)	78	11.3%
Bickhart et al., 2012 [36]*	Resequencing	3	5	1032	41.1 Mb (1.6%)	128	12.4%
Ben Sassi et al., 2016 [61]	Resequencing	1	10	823	45.4 Mb (1.8%)	90	10.9%
Choi et al., 2016 [62]	Resequencing	2	20	901	5.5 Mb (0.22%)	137	15.2%
Gao et al., 2017 [16]	Resequencing	1	8	400	6.9 Mb (0.27%)	36	9%
Present study	BovineHD	1	215	1066	181.6 Mb (7.2%)	–	–

Based on the bovine genome assembly UMD_3.1, considering only autosomal CNVRs

*: CNVRs was mapped to the Btau_4.0 genome builds in the published paper

50 K-Illumina: BovineSNP50 BeadChip

BovineHD: Illumina BovineHD BeadChip

CGH: Comparative Genomic Hybridization

A total of 65 CNVRs were identified as being yak-specific CNVRs (Additional file 8: Table S8), after discarding the CNVRs reported by the 31 previous studies. These yak-specific or novel CNVRs included 4 gains, 59 losses, and 1 gain/loss event.

Forty-two of the 65 identified yak-specific CNVRs were annotated, containing a total of 70 genes (Additional file 9: Table S9). Functional annotation and enrichment showed that the genes harbored in these regions were significantly enriched in biological processes associated with transmembrane receptor protein tyrosine kinase signaling pathways, mesoderm development, the MAPK cascade, the regulation of molecular functions, and phosphate-containing compound metabolic processes. An additional PANTHER pathway analysis revealed that the main pathways enriched in these genes included the Ras pathway, angiogenesis, and the gonadotropin-releasing hormone receptor pathway (Additional file 10: Table S10). A total of 9 genes out of the 49 named protein coding genes in this gene set were found to be associated with hypoxic stress (Additional file 9: Table S9).

38 of the 65 yak-specific CNVRs harbored 106 QTLs associated with exterior traits, health traits, meat and carcass traits, milk traits, production traits, and reproduction traits (Additional file 11: Table S11).

## Discussion

Genetic variations are vital sources of phenotypic variations associated with various traits that may be advantageous in different contexts. CNVs are a major source of genetic variation in livestock genomes, potentially affecting many economically important traits such as growth traits [5, 12, 24], meat quality traits [18, 19, 22], milk production [15], disease resistance [11, 63], and coat color [21]. Yaks live in an extremely harsh environment in QTP, and are well adapted to the low levels of oxygen, high levels of UV radiation, and cold temperatures endemic to this region. As such they represent an ideal organism for the study of mechanisms of high altitude adaptation. Previous studies of CNVs have revealed that CNVs across the genome may be linked to key environmental adaptations [64–67]. Compared to other livestock, genome-wide CNV maps for the yak genome remain limited, with just one currently being available [27].

In the present study we therefore generated the first genome-wide yak CNV map based on the Illumina BovineHD Beadchip, with the goal of assisting in yak molecular breeding and gaining new insights into the genetic architecture underlying high elevation adaptations in this species.

### CNVRs detected in the yak genome

A total of 1066 CNVRs were successfully detected across the yak autosomal genome, with a total length of 181.6 Mb, covering ~ 7.2% of the genome. These CNVRs ranged in size from 5.53 kb to 1148.45 kb, with an average size of 170.31 kb. Eight out of the nine randomly selected CNVRs were successfully validated by the qPCR experiment. The failure to validate the ninth CNVR may be due to inadequately designed qPCR primers used to confirm the CNVRs, which may have targeted an area outside of the true CNVR, because of the failure to accurately identify the real CNVR breakpoints by *PennCNV* [32].

In a previous report, 2634 CNVRs were discovered in the yak genome with a total length of 153 Mb, covering ~ 5.7% of the yak genome [27]. In this previous study, the size of these CNVRs ranged from 3 kb to 1309 kb with an average size of 58 kb.

The number of CNVRs identified in the present study is substantially lower than in the previous study. The total length and average size of the CNVRs in the present study, however, were actually larger than in the previous report. There are some reasons which may account for the differences in the CNVR results between the two studies. First of all, the CNV detection parameter of previous study was set to > 1.5 kb, whereas in present study, only the CNVs longer than 50 k were targeted to reduce the risk of a high false positive rate according to the user guide for the *PennCNV* software. This difference in filter criteria during the first step of the CNV detection may largely affect the CNVs available for CNVR definition, which may in turn have led to the differences in both the number of CNVRs and their length. Secondly, the different platforms used for CNV calling and SNP discovery may have impacted study outcomes. Here, we generated a CNVR map based on a SNP array, while the previous study conducted resequencing to infer CNVRs. In general, CNVRs detected via SNP array are longer than those identified by resequencing as a consequence of the poorer genomic coverage of such SNP arrays [16, 41]. Even though our present study used a high density chip-based SNP array, the coverage was less than that offered by resequencing. Thirdly, the algorithms used to detect the CNVs also differed between these two studies. In the previous report, NGS data were used for CNV detection and the CNV calling program adopted was *CNVnator,* which uses Read-depth (RD) analysis algorithms [68]. In the present study, the software *PennCNV* was employed. *PennCNV* is a hidden Markov model-based (HMM) algorithm that uses the log R Ratio (LRR) and B Allele Frequency (BAF) in combination [69]. Fourth, the methods used to define the CNVRs were different. In this study a freely available program named *CNVRuler* [42] was used, while in the previous study a custom Perl script was adopted. The detailed information of parameters used for defining CNVRs of the previous study was not available. Here, we trimmed the sparse area by using the regional density (recurrence) threshold. Regions of low density of overlapping (recurrence < 0.3) were not used in the analyses for a more robust definition of the beginning and end of regions. In addition, the different genetic background of the animals tested in these two studies may further contribute to this difference. A report in cattle showed that the number of CNVRs detected in a population consisting of multiple breeds may be larger than that in a population including only one breed [41]. In the present study, all samples were from one yak breed, whereas previous study tested 14 wild yaks and 65 domestic yaks from various locations across the QTP.

Given the limitations in terms of available genome-wide yak CNVR maps, we also compared our results to the numerous studies involving other bovine species such as cattle, zebu, and buffalo (Table 3). We found substantial differences in identified CNVRs among these studies, likely linked with differences in breeds, sample numbers, platforms used for CNV detection, and CNV calling algorithms used in each individual study. Four primary platforms are used for bovine genome assessment: CGH array, Illumina BovineSNP50, Illumina BovineHD BeadChip, and Resequencing. In previous reports, these different approaches were used in 6, 6, 14, and 5 studies, respectively. The differences between the three primary SNP array-based platforms are primarily centered on their relative ability to detect small CNVs in the genome [52]. A previously study compared CNVRs detected using the Illumina BovineSNP50 and Illumina BovineHD platforms, determining that CNVRs identified on the basis of low density chips were longer than those based on high density chips, emphasizing the importance of genome coverage to CNVR length determination [41]. Apart from the platform employed, the software and underlying algorithms likely resulted in substantial variations in results among the aforementioned 31 studies. In addition to *PennCNV*, there are several other software packages including *QuantiSNP* [70] and *cnvPartition* (Illumina) commonly used for CNV detection. Each algorithm has its own unique strengths and weaknesses [71], and these may influence results of CNV detection efforts. In the 31 listed studies, the software used to detect CNVs across the genome included *PennCNV*, *cnvPartition, LUMPY* [72]*,* and *GADA* [73]*.*

### CNVR functional enrichment analyses reveal the potential molecular adaptations to high altitudes

The gene ontology (GO) analysis of the genes harbored in the identified CNVRs showed that they were significantly enriched for functions including sensory perception of smell and chemical stimuli (GO: 0007608 and GO: 0007606), lipid metabolism (GO: 0006869, GO: 0006633 and GO: 0006631), responses to stimuli (GO: 0050896), cellular processes (GO: 0009987), G-protein coupled receptor signaling (GO: 0007186), and JAK-STAT signaling (GO: 0007259). When we mapped the CNVRs-associated genes onto the Reactome pathway, our results confirmed that these genes were involved in olfactory signaling (R-BTA-217271) and lipid metabolism (R-BTA-1483257, R-BTA-1483191, R-BTA-77289 and R-BTA-804914). Additionally, this Reactome pathway analysis showed that these CNVRs were enriched for genes participating in prolactin receptor signaling (R-BTA-1170546), growth hormone receptor signaling (R-BTA-982772), FMO oxidation of nucleophiles (R-BTA-217271), and digestion and absorption (R-BTA-8963743).

Among these analyses, the most significantly enriched pathway was the olfactory signaling pathway, and CNVs in genes within this pathway have been found in many previous reports across many species, including cattle [18, 31], yak [27], sheep [37], dog [74], pig [75], rat [76], horse [40] and human [77]. This gene family thus varies widely between mammalian species, with evidence of ongoing evolutionary changes resulting in novel genes and gene activities – changes in which CNVs are likely to play a substantial role [78]. The identified sensory perception GO categories are of great importance for allowing organisms to effectively respond to the environment, and a comparative genome study has previously revealed that gene families relating to sensory perception in yaks are substantially expanded as compared with cattle [26]. Unlike in the cattle industry, yaks are raised in pastures without any concentrated supplement being provided for nutritive purposes. Given a previous study indicating that yaks grazed late into the night during transitional and summer seasons in pastures [79], CNVs in olfactory signaling genes may allow them to better obtain food at night. Such changes in sensory perception and olfactory signaling may thus be involved in the high elevation adaptations of yaks.

Another significantly enriched functional category among our CNVRs pertained to lipid metabolism, with detectable enrichments in genes associated with lipid transport (GO: 0006869), synthesis of phosphatidylcholine (R-BTA-1483191), fatty beta-oxidation (R-BTA-77289), and fatty acid biosynthesis (GO: 0006633). Interestingly, the function of genes relating to lipid metabolism has been found to be enriched among positive selected genes in the yak [26] and ground tit genomes [80]. Positively selected genes in animals living at high elevations may thus play an important role in the adaptation to this harsh environment, where animals are faced with low oxygen, high levels of UV radiation, and cold temperatures. Creatures living in such harsh environments must be able to sustain thermogenesis in order to survive. Lipid metabolism-related functions can enable animals, including yaks, to produce enough energy under conditions of cold temperature stress [80]. One study specifically found that the n-3 polyunsaturated fatty acid present in phosphatidylcholine may improve the ischemic tolerance of chronically hypoxic heart tissue [81]. As we observed a functional enrichment in the pathway associated with phosphatidylcholine synthesis among genes in CNV gain regions, these regions may thus be specifically linked to the high altitude adaptation of yaks.

Plant resources in high altitude regions are not only limited, but are also of low quality owing to the harsh environmental conditions. Among genes in regions associated with CNV gain events, the digestion and absorption pathway (R-BTA-8963743) was significantly enriched, which may be related to the ability of yaks to cope with the situation of forage shortage.

We were particularly interested in the biochemical characteristics of the “FMO oxidizes nucleophiles” pathway (R-BTA-217271). Flavin-containing-monooxygenases (FMO) can catalyze the NADPH-dependent N- or S-oxygenation of heteroatom-containing compounds. FMO is thought to have evolved as a xenobiotic detoxification catalyst to protect mammals from lipophilic nucleophilic plant chemicals in early environments [82]. Plants grown under abiotic stress conditions, such as those grown in cold conditions, can accumulate many phenolic secondary compounds [83], which may have toxic effects in mammals. Yaks lives in QTP feeding on plants containing high phenol levels, so CNVs in genes linked with FMO nucleophile oxidation may thus be valuable in detoxifying these plant chemicals.

To better explore the molecular adaptations of yaks to high altitude conditions, we conducted a functional analysis of the gene sets associated with yak-specific CNVRs. A GO analysis showed that genes in these yak-specific CNVRs were significantly enriched for biological processes involved in phosphate-containing compound metabolism (GO: 0006796), MAPK signaling (GO: 0000165), regulation of molecular functions (GO: 0065009), transmembrane receptor protein tyrosine kinase signaling (GO: 0007169), and mesoderm development (GO: 0007498). When mapping the genes onto PANTHER pathways, we identified significant enrichments for the gonadotropin-releasing hormone receptor pathway (P06664), angiogenesis (P00005), and the Ras pathway (P04393) in this gene set.

The GO term “phosphate-containing compound metabolic process” (GO: 0006796) was significantly enriched among positively selected genes involved in the hypoxia response in the ground tit [80]. Among the three pathways identified in the present study, angiogenesis (P00005) is well known to be an important process involved in responding to hypoxia by increasing the oxygen supply, a function that has also been observed in other high-elevation animals [84, 85].

### QTLs harbored in yak CNVRs are associated with economically important traits

Previous studies have reported that many CNVRs harbor QTLs associated with economically important traits in cattle [15, 18, 19]. In this study, we therefore examined the CNVRs in the cattle QTL database, identifying a total of 1736 QTLs involved in six classes of traits (Additional file 7: Table S7) within 396 CNVRs. Affected traits included those associated with reproduction QTLs, production QTLs, meat and carcass QTLs, milk QTLs, exterior QTLs, and health QTLs. In the 65 yak-specific CNVRs, 106 QTLs were mapped to 38 CNVRs.

On the QTP, yaks are an essential source of meat for high-altitude societies, making the genetic improvement of traits pertaining to meat production and quality to be of great importance in the context of yak breeding.

Of the identified QTLs pertaining to meat and carcass quality, both intramuscular fat and shear force are considered to be two major traits that are the focus of improving meat quality in the yak industry. We found *IRAK3* to be one identified candidate gene in a QTL locus associated with intramuscular fat levels. In cattle, the interleukin-1 receptor-associated kinase 1 (*IRAK1*) gene has been found to be differentially expressed in skeletal muscle samples containing different amounts of intramuscular fat, but the underlying mechanistic link remains uncertain [86]. The *IRAK3* gene, which shares substantial homology with *IRAK1*, may therefore also participate in yak intramuscular fat deposition, making it a viable candidate gene for future studies aimed at improving meat quality. With regard to sheer force, *MUSK*, *NRXN1,* and *TBC1D4* were identified as being candidate genes in the identified QTL loci in this study. Among these, muscle-associated receptor tyrosine kinase (*MUSK*) plays a central role in the formation and the maintenance of the neuromuscular junctions (NMJ) - the synapses between motor neurons and skeletal muscle. It is expressed in embryonic muscle [87], and mutations in this gene can cause fetal akinesia deformations [88]. The mechanisms by which this gene affect the sheer force of meat remains unknown, making it another viable candidate gene for further investigation aimed at improving meat quality.

## Conclusions

Here, we produced the first genome-wide CNV map of yak based on the Illumina BovineHD BeadChip genotype data from 215 polled yaks. A total of 1066 CNVRs were found in the yak genome, with a total length of 181.6 Mb, comprising ~ 7.2% of the bovine autosomal genome. Functional enrichment analyses of the genes present within these CNVRs revealed a number of potential molecular adaptations that may enable yak to live in its harsh high elevation habitat. QTL detection showed that more than one third of CNVRs overlapped with QTLs associated with economically important traits in yak. The results found here may therefore provide valuable insights into the molecular mechanisms of high altitude adaptation as well as the potential genomic basis of economically important traits in yak。.

In the future, at least two types of studies will be needed to explore the potential value of CNVs in the yak genome. First, CNV-based genome-wide association studies including more polled yaks are required to evaluate the potential effect of these CNVs on the formation of economically important traits related to growth, meat production, milk production, reproduction, and health in these yak. Identification of genetic loci responsible for disease resistance based on CNV can somehow benefit the yak welfare by avoiding them suffering from the disease. In addition, SNP array data from additional yak breeds as well as other bovine species such as cattle, zebu, and buffalo should be collected and used to generate a comprehensive genome-wide bovine CNV map, which can offer value to the investigation of population genetics based on CNVs.

## Methods

### Sample collection and genotyping

The 215 experimental animals were young female polled yaks born in 2017 and originating from Datong yak farm, Datong County, Xining, Qinghai province of the People’s Republic of China. Blood samples were collected from all animals. Genomic DNA was extracted from these blood samples using the EasyPure Blood Genomic DNA Kit (TransGen Biotech Co., LTD, Beijing, China). The concentration and purity of DNA were measured using a NANODROP 2000 Spectrophotometer (Thermo Fisher Scientific Inc., Waltham, MA USA).

The Illumina BovineHD BeadChip (Illumina Inc., CA, USA) was used to genotype the animals. The Illumina BovineHD BeadChip is the most comprehensive genome-wide genotyping array available for bovine species. It contains 777,962 SNPs uniformly spanning the bovine genome with a 3.43 kb mean genomic coverage.

### Genome-wide detection of CNVs and CNVRs

The *PennCNV* software [69] was utilized to identify the CNVs in the yak genome. The *GenomeStudio* program was used to generate the input signal intensity file containing the information needed to call CNVs, including SNP name, chromosome, position, genotype, Log R Ratio (LRR), and B Allele Frequency (BAF). The PFB file was generated by running the perl script ‘compile_pfb.pl’ provided by the *PennCNV* program based on the average BAF of each marker in the population.

After all required files were prepared, the script ‘detect_cnv.pl’ was used to detect the CNVs of all individuals. The ‘-lastchr 29’ argument was used to declare that the last autosomal chromosome as being 29 (rather than 22). To eliminate the impact of genomic waves on the CNV calling procedure, the GC model file, which was generated by calculating the 1 Mb genomic regions surrounding each marker (500 K on each side), was used as the parameter of the option ‘-gcmodel’. The raw CNV calls were filtered using the perl script ‘filter_cnv.pl’ with the filter criteria: SNP number > 10 and CNV length > 50 k.

The *CNVRuler* software was then used to merge the overlapping CNVs identified across all the samples. There are three methods provided by *CNVRuler* to define the CNVRs, namely CNV region (CNVR), Reciprocal Overlap (RO), and Fragment. Here we chose the CNVR method. The value of recurrence was set to 0.3 to trim sparse areas of the overlapping regions in order to avoid overestimating the size and frequency of CNVRs.

### Validation of CNVRs by qPCR

Experimental validation of identified CNVRs was performed by qPCR using a CFX96 Real-Time System. Nine CNVRs identified by *CNVRluer* were chosen at random based on the CNV type (loss, gain, and both gain/loss) and frequency. The primers (Additional file 12: Table S12) were designed using the Primer3 webtool (https://www.ncbi.nlm.nih.gov/tools/primer-blast) based on UMD_3.1 genome assembly. The genomic DNA samples used for SNP genotyping were then also used for qPCR validation. The bovine basic transcription factor 3 gene (*BTF3*) was used as reference gene as previously reported [5]. For each CNVR, six samples with copy number variations and one normal copy sample were chosen based on the CNV detection results from the *PennCNV* software to validate the inferred CNVRs.

qPCR experiments were conducted using the TB Green Premix Ex Taq PCR Reagent Kit (Takara Bio). qPCR was carried out in a total volume of 20 μL and the reaction conditions were as follows: 95 °C for 3 min, followed by 40 cycles for 95 °C for 10 s, 60 °C for 10 s and 72 °C for 10 s. Three replicate reactions were performed for each sample. The 2^−ΔΔCt^ method was used to calculate the fold changes, and 2 × 2^−ΔΔCt^ was calculated as the copy number of the target genes in the test samples.

### Gene detection and functional analysis

Genes harbored in or partially overlapping with CNVRs were retrieved from the Ensembl Genes 93 Database using BioMart (http://useast.ensembl.org/biomart/martview/c2108f4dd2c5515aa38f7e8a4696d95c). Functional enrichment analyses of the genes overlapping with specific CNVRs were performed using tools offered by the Gene Ontology Consortium (http://www.geneontology.org/).

Named protein coding genes were queried against to the list of hypoxia-related genes from the HypoxiaDB database [44] in order to identify the genes involved in hypoxic response harbored in yak CNVRs.

### QTL detection within CNVRs

To investigate the potential associations between CNVRs and economically important traits in yaks, the Animal QTL database (https://www.animalgenome.org/cgi-bin/QTLdb/BT/index) was employed to map the Cattle QTL loci to these CNVRs.

### Comparison with previous studies

To identify yak-specific CNVRs, we compared our results with those of previous studies involving many different breeds of cattle, zebu, and buffalo. As some of these previous results were mapped on the Btau_4.0 genome builds, we converted those results from Btau_4.0 to UMD 3.1 using the LiftOver tool (https://genome.ucsc.edu/cgi-bin/hgLiftOver) on UCSC before comparison. The CNVRs on non-autosomal chromosomes or in unplaced locations were also excluded before analysis. The yak-related CNVRs in the results of the study conducted by Zhang et al. [46] were discarded prior to comparison.

Then, functional enrichment analyses and QTL detection were performed on the yak-specific CNVRs.

## Additional files

Additional file 1: **Table S1.** Detail information about raw CNVs calling results. (XLSX 1726 kb)

Additional file 2: **Table S2.** Detail information about CNVs after filtering. (XLSX 1227 kb)

Additional file 3: **Table S3.** Detail information of the detected CNVRs. (XLSX 59 kb)

Additional file 4: **Table S4.** Information about Ensembl genes found to be overlapped with CNVR identified in the present study. (XLSX 134 kb)

Additional file 5: **Table S5.** Significantly enriched GO terms and Reactome pathways of genes associated with the identified CNVRs. (XLSX 11 kb)

Additional file 6: **Table S6.** Hypoxia-related genes found to be overlapped with CNVRs identified in the present study. (XLSX 16 kb)

Additional file 7: **Table S7.** Information about QTLs found to be overlapped with CNVRs identified in the present study. (XLSX 104 kb)

Additional file 8: **Table S8.** Detail information of the yak-specific CNVRs. (XLSX 11 kb)

Additional file 9: **Table S9.** Information about Ensembl genes found to be overlapped with yak-specific CNVRs identified in the present study. (XLSX 14 kb)

Additional file 10: **Table S10.** Significantly enriched GO terms and PANTHER pathways of genes associated with the yak-specific CNVRs. (XLSX 9 kb)

Additional file 11: **Table S11.** Information about QTLs found to be overlapped with yak-specific CNVRs identified in the present study. (XLSX 16 kb)

Additional file 12: **Table S12.** Detail information about primers used for CNVRs validation. (XLSX 9 kb)

## Abbreviations

BAF: B Allele Frequency
CGH: Comparative genomic hybridization
CNV: Copy number variation
CNVR: Copy number variation region
FoSTeS: Fork stalling and template switching
GO: Gene Ontology
HMM: Hidden Markov Model
LRR: Log R Ratio
NAHR: Non-allelic homologous recombination
NGS: Next-generation sequencing
NHEJ: Non-homologous end-joining repair of double-stranded breaks
qPCR: Real-time Quantitative Polymerase Chain Reaction
QTL: Quantitative trait locus
QTP: Qinghai-Tibetan Plateau
RD: Read-depth
SNP: Single nucleotide polymorphisms
